# Results of a Digital Multimodal Motivational and Educational Program as Follow-Up Care for Former Cardiac Rehabilitation Patients: Randomized Controlled Trial

**DOI:** 10.2196/57960

**Published:** 2024-12-11

**Authors:** Maxi Pia Bretschneider, Wolfgang Mayer-Berger, Jens Weine, Lena Roth, Peter E H Schwarz, Franz Petermann

**Affiliations:** 1 Department for Prevention and Care of Diabetes, Department of Medicine III, Faculty of Medicine Carl Gustav Carus, Technische Universität Dresden, Fetscherstraße 74, Dresden, 01307, Germany, 49 351 458 2715; 2 Klinik Roderbirken der Deutschen Rentenversicherung Rheinland, Leichlingen, Germany; 3 Vision2B GmbH, Erfurt, Germany

**Keywords:** mHealth, apps, digital technology, digital interventions, coronary heart disease, lifestyle intervention, cardiac rehabilitation, quality of life, cardiac care

## Abstract

**Background:**

Digital interventions are promising additions for both usual care and rehabilitation. Evidence and studies for the latter, however, are still rare.

**Objective:**

The aim of the study was to examine the app/web-based patient education program called “mebix” (previously called “Vision 2 – Gesundes Herz”) regarding its effectiveness in relation to the parameters of disease-specific quality of life (HeartQoL), cardiovascular risk profile (Cardiovascular Risk Management [CARRISMA]), and prognostic estimation of early retirement (Screening instrument work and occupation [SIBAR]) in 190 participants from a cardiological rehabilitation clinic.

**Methods:**

To evaluate mebix, 354 patients from the Roderbirken Clinic of the German Pension Insurance Rhineland (Germany) with a coronary heart diesase were recruited and randomized either to the intervention group (using mebix postrehabiliation for up to 12 months) or the control group (receiving standard care). The data collection took place at the end of inpatient rehabilitation (t0), as well as 6 months (t1) and 12 months (t2) after the end of rehabilitation. Analyses of variance are used to assess the overall significance of difference in outcome parameters between groups and over time.

**Results:**

The primary endpoint of disease-related quality of life shows a significant improvement of 7.35 points over the course of the intervention that is also more pronounced in the intervention group. Similarly, the 10-year risk of cardiovascular death and myocardial infarction showed significant improvements in the cardiovascular risk profile over time and between groups, indicating better results in the intervention group (ie, a reduction of −1.59 and −5.03, respectively). Secondary outcomes like the body weight and cholesterol levels were significantly reduced in the intervention group, also in comparison with the control group. In addition, the SIBAR was significantly lower/better at the end of the observation period than at the beginning of the observation for both groups.

**Conclusions:**

Overall, the digital training program represents an effective follow-up offer after rehabilitation that could be incorporated into standard care to further improve disease-related quality of life and cardiovascular risk profiles.

## Introduction

Cardiovascular diseases (CVD) are the most common cause of death worldwide and result in not only serious health impairments but also significant health care costs [1]. In Germany, the Gesundheit in Deutschland aktuell (GEDA) 2014/2015-European Health Interview Survey (EHIS) study found a 12-month prevalence of coronary heart disease of 3.7% in women and 6.0% in men, which increases with age [2]. The follow-up study from 2019/2020 showed an increase for both genders (women: 5.1%; men: 6.6%) [3]. CVDs account for the largest share of health care costs in Germany [4]. The existence of cardiologically relevant risk factors in the population is undisputed. For example, in the large-scale EUROASPIRE study, modified risk factors were analyzed in 4863 patients with coronary heart disease (CHD) based on hospital reports and medical examinations [5,6]. The results showed that 19% of patients smoked, 25% had a BMI ≥30, more than half (53%) had elevated blood pressure (systolic blood pressure ≥140 mm Hg and/or diastolic blood pressure ≥90 mm Hg), 44% had elevated cholesterol (>5.5 mmol/L), and 18% had diabetes mellitus (HbA_1c_ >48 mmol/mol; 6.5%). Half of the patients taking antihypertensive medication had high blood pressure (systolic >140 mm Hg; 21% >160 mm Hg). Of the patients taking lipid-lowering medication, 49% had elevated cholesterol levels (>5.5 mmol/L and 13% >6.5 mmol/L). In 37% of the patients, a family history of CHD was present.

In medical rehabilitation in general and in cardiological rehabilitation in particular, secondary preventive treatment modules should aim at reducing cardiovascular risk factors and supporting health-promoting behaviors. Thus, professional reintegration and rehabilitation and increasing quality of life are of particular importance. However, the behavioral and attitudinal changes taught for this purpose during rehabilitation, such as favorable exercise or dietary habits leading to an increased quality of life, are difficult to establish and sustain for many patients in daily life.

Although health care guidelines place a high priority on the further development and evaluation of aftercare concepts to maintain and improve what has been achieved in rehabilitation [7], the potential for secondary prevention measures in patients with CHD is far from being exhausted and some existing offers have not been able to achieve the desired outcomes [5,6,8,9].

So far, only a few studies have investigated how this transfer can be effectively supported in the long term. According to a review, there are generally positive effects for the patient education measure in cardiology [10]. A controlled study with cardiovascular rehabilitation patients that included telephone follow-up over 36 months showed a positive effect on their cardiovascular risk profile, disease-related quality of life, and morbidity (disability pensions) [11]. However, in the EUROASPIRE study, only cholesterol levels were favorably influenced in the patients.

Meta-analyses have shown that digital applications can positively influence the risk factors of CHD and therefore also represent patient-centered secondary prevention [12-14]. Studies that investigate the impact of new technologies (such as SMS text messaging, email, smartphones, internet chat, online coaching, and web diaries) on cardiovascular follow-up rarely focused on training programs in the form of infotainment (DVDs or video streaming) combined with online support and a reminder service involving partners [15]. Moreover, most digital health interventions focus on physical counseling and exercise training, leaving out other core components for cardiovascular rehabilitation [16]. In Germany, a novel app/web-based patient education program, “mebix,” previously called “Vision 2 — Gesundes Herz,” was developed by a multidisciplinary team under the patronage of the German Society for Prevention and Rehabilitation of Cardiovascular Diseases (DGPR). It aims to offer patients a digital health intervention that incorporates all relevant components for cardiovascular rehabilitation.

The primary objective of this study is to evaluate mebix as a new and innovative form of patient education as a cardiological follow-up intervention. The main aim is to determine whether mebix is associated with an impact on disease-specific quality of life. Further objectives include effectiveness for improving cardiovascular risk profile and prognostic estimation of early retirement.

## Methods

### Ethical Considerations

The study was conducted in accordance with the Declaration of Helsinki and approved by the Ethics Committee of the University Bremen on May 5, 2015. All participants were informed about the study and asked to participate. Patients were informed verbally and in written form that participation in the study was voluntary and that withdrawing their informed consent was possible at any time without giving reasons. The participants received no compensation for expenses. The data was pseudonymized, that is, the identification data for a specific person (eg, name, insurance number) was replaced by an identification number, to avoid or hinder the identification of the person.

### Study Procedures

The study was carried out by the Centre for Clinical Psychology and Rehabilitation at the University of Bremen from February 2015 to June 2019 at the Roderbirken Clinic of the German Pension Insurance Rhineland. The design is based on a randomized prospective controlled trial and is registered in the German Register of Clinical Studies (DRKS00007569).

The patients received an introduction to the mebix program at the end of their inpatient stay in the rehabilitation facility by previously trained clinic staff. The participants only received a DVD box with 2 DVDs and a booklet containing instructions on how to carry out the program. The booklet also contained the online key (password) with which the participants could log in to the online portal/app. At home, the participants carried out the training over a period of approximately 4‐12 weeks (with online follow-up for 1 year).

Data collection involved questionnaires at 3 time points: at baseline in the clinic (t0) and after 6 months (t1) and 12 months (t2). The medical data required for the cardiological risk profile at the time of measurement t1 and t2 were collected from the patient’s general practitioner or specialist. The corresponding questionnaire was filled out by the attending physician and sent to the study center by the patient.

### Recruitment

Participants were recruited at the Roderbirken Clinic of the German Pension Insurance Rhineland. All newly admitted patients who met the inclusion criteria (Textbox 1) were informed about the study and asked to participate. Patients were informed verbally and in written form that participation in the study was voluntary and that withdrawing the previously given written informed consent was possible at any time without giving reasons. The ability to give consent was checked based on the inclusion and exclusion criteria (physician’s judgment). No minors or incapacitated adults were included in the study. When eligibility was not confirmed, participants were excluded from the study. When patients fulfilled the inclusion and exclusion criteria, they were randomized by study personnel to either the intervention or control group. For this, block randomization was used.

A standardized information event was developed for the patients, including an information film and an information flyer. From July 1, 2015, the recruitment of the study patients began in the clinic, as did the regular implementation of information events for the patients and data collection (t0). The information events for the patients for the purpose of recruitment initially took place weekly, then fortnightly from March 1, 2016. The patients were recruited until December 31, 2017.

Textbox 1. **Inclusion and exclusion criteria.**

**Inclusion criteria**

- Patients of the German Pension Insurance Rhineland who are at the end of inpatient cardiological rehabilitation
- Age ≤60 years
- Confirmed coronary heart disease
- Sufficient knowledge of German, reading and writing ability
- Availability of a PC and online access
- Signed informed consent

**Exclusion criteria**

- Severe prognosis-limiting factors (heart failure, New York Heart Association Class III and IV)
- Severe chronic obstructive pulmonary disease (forced expiratory volume [FEV] <35%, respiratory global insufficiency, chronic inflammation, consumptive disease). FEV describes the air that is exhaled in 1 second and is used to measure chronic pulmonary disease and its progression. A FEV1 below 35% indicates very severe disease.
- Cognitive or language impairment
- Lack of informed consent

### Intervention

Both intervention and control group patients received usual care after the end of the rehabilitation and were free to participate in outpatient services. Control group patients received a written summary of important information on a healthy lifestyle (diet, exercise, etc). The intervention group, on the other hand, received access to the app/web-based patient education program mebix.

The media package was developed under the patronage of the DGPR with leading cardiologists, sports physicians, and metabolism experts, and is based on the latest medical findings consisting of the following modules:

1. Coronary heart disease
2. Successful therapy
3. Heart-healthy nutrition
4. How to get moving or how to get moving safely and without fear
5. Finally smoke-free
6. High blood pressure
7. Heart attack and rehabilitation
8. Heart failure and cardiovascular arrhythmias
9. Tips for everyday life
10. Nordic walking
11. Stress relief

Over a period of approximately 4-12 weeks, the training involved 11 films/modules that built on each other. After each film/module, users could check their acquired knowledge in a multiple-choice test on the associated personalized online portal/app. The system then evaluated the answers so that users could see which questions had been answered correctly or incorrectly. Users then had the option of repeating the test until all questions were answered correctly. The practice tasks within the digital intervention include instructions with concrete objectives and accompany the participants over a period of 1 year. They help the participant to reflect on the interactions between lifestyle and health status (eating habits, exercise, risk of secondary diseases, etc) and to develop a healthier lifestyle (eg, increase physical exercise and eat a healthier diet).

Tools such as a diet and exercise log (energy balance calculator) make recommendations and values less abstract, making it easier for the patient to approach dietary and weight recommendations in everyday life. The exercises support all phases that patients must go through in a successful training: the phases of reflection (recognize risk) and activity (change lifestyle). The exercise units are a self-structured sequence and not, like the knowledge test, thematically bound to modules. The values from the practical exercises (eg, recovery pulse, resting pulse) enable patients to gain direct insight into their therapy success. The reminder service via email or SMS text messaging informs or reminds users according to the progress of the training and the tasks to be completed (eg, if the knowledge test or the nutrition and exercise protocol has not been completed). An example of the user interface of mebix can be found in Multimedia Appendix 1.

### Statistical Analysis

#### Outcomes and Analysis

The primary outcomes of this study are the disease-specific quality of life, which is measured by the HeartQoL quality of life questionnaire for CHD patients [17], and body weight. The HeartQoL uses a 4-point scale to measure the extent to which everyday activities and physical (10 items) and emotional (4 items) functioning are impaired by CHD. By adding up the item values, a total score (0‐42 points) and scores for physical (0‐30 points) and emotional (0‐12 points) quality of life are obtained, with higher values indicating a higher quality of life. An improvement in disease-specific quality of life of 0.3 in the HeartQoL total score is considered clinically significant.

Secondary outcomes are the reduction of the cardiovascular risk profile and the improvement of the employment prognosis or the number of participants on disability pensions. The cardiovascular risk profile is determined by a web-based program called Cardiovascular Risk Management (CARRISMA) in primary prevention [18]. The CARRISMA program includes the patient’s personal data, information on body composition (weight, height, BMI), smoking behavior, family history of CHD, and medications. Furthermore, the following medical parameters can be recorded: blood pressure, blood lipids, carbohydrate metabolism (HbA_1c_, fasting blood sugar), and kidney values, as well as previous diagnoses or events (eg, heart failure, bypass surgery) and additional risk factors. In addition, information on activity (calculation of weekly calories burned due to preferred activities) and diet (calculation of weekly calorie consumption via preferred foods and beverages) can be entered. CARRISMA calculates the cardiovascular risk profile on the basis of the patient data entered (10-year risk of cardiovascular death and 10-year risk of myocardial infarction) in the form of known scores (ie, European Society of Cardiology [ESC] score, Prospective Cardiovascular Münster [PROCAM] Study, Framingham). People who do not yet fall into the range requiring treatment with the conventional scores have a significantly higher risk of CVD when obesity and heavy cigarette consumption are considered. In the CARRISMA, this effect is considered in addition to the results of the risk assessment of the ESC score, PROCAM, and Framingham, as well as the result for the 3 scores with and without the additional consideration of these lifestyle factors. Information from 2 data sources is entered into the CARRISMA program:

- The medical parameters questionnaire (redesign) collects all relevant medical data, for example, diagnosis, duration of illness, and laboratory and examination results (blood pressure, total cholesterol, HbA_1c_, body weight).
- The questionnaire on activity and dietary behavior (new construction) records the amount of physical activity and the choice of food.

The “Screening instrument work and occupation” (SIBAR) tool is also used for primary prevention to record current employment status, the degree of reduced earning capacity, and pension entitlement [19]. Cutoff values are available that indicate an increased sociomedical risk of early retirement and the perceived degree of stress of patients with regard to their occupational situation. The SIBAR is intended to be a data-based tool for assessing the need for occupational treatment services. These requirements result in three subscales of the SIBAR:

1. Sociomedical/risk of early retirement: With the help of this scale, the subsequent application behavior for early retirement for health reasons is predicted (value range 0‐19). A significantly increased risk of early retirement and a need for occupational treatment exist with a score of at least 8.
2. Occupational stress: An indication for specific occupational measures arises if rehabilitants subjectively describe their occupational situation as highly stressful overall (value range 0‐1).
3. Subjective need for occupation-related treatment (value range 0‐1).

All 3 scales are added together for the overall SIBAR index. An indication of a need in the respective scale is counted as “1,” and no need is counted as “0.” This results in an overall SIBAR score of 0‐3 points. The authors assume that there is a need for work-related treatment services if there is an overall SIBAR score of at least 2.

Table 1 demonstrates an overview of the outcome measurements at different time points.

The results for both the 6- and 12-month follow-up will be summarized using descriptive statistics. For the primary analysis to assess group differences, a 2-factor ANOVA (ie, including time, group, and their interaction as the main factor) will be used. In case of a significant main effect of the group and time interaction, significant group effects can be assumed. Additionally, exploratory paired *t* tests will be used to assess the pre-post effects of mebix on the intervention group at 6 months and to quantify the effectiveness of the intervention.

All statistical analyses were performed using IBM SPSS Statistics (version 20; IBM Corp).

**Table 1.** Overview of outcome measurements at different time points.

	Completion of rehabilitation (t0)	Six months after completion of rehabilitation (t1)	Twelve months after completion of rehabilitation (t2)
**Sociodemographic data**	X		
**Disease-related quality-of-life questionnaire (HeartQoL)**	X	X	X
**Cardiovascular risk profile by Cardiovascular Risk Management (CARRISMA)** **program**			
Medical parameters questionnaire	X	X	X
Questionnaire on activity and dietary behavior	X	X	X
**Screening instrument work and occupation (SIBAR)**	X	X	X

#### Power

The target sample size was based on a 2-tailed *t* test with a power of 80% and an α of 5%. The number of participants needed to detect a difference with a small to medium effect size (Cohen *d*) of 0.3 was 175 per group. With an assumed dropout rate of 40%, the required sample size was 250 per group.

#### Data Exclusion

All analyses were conducted according to the intention-to-treat principle, that is, all participants were analyzed as randomized. Missing data points were imputed using the last observation carried forward method, that is, the last available data point was used for the missing data point(s). A *P* value <.05 was considered statistically significant.

## Results

### Participant Characteristics

**Figure 1.**
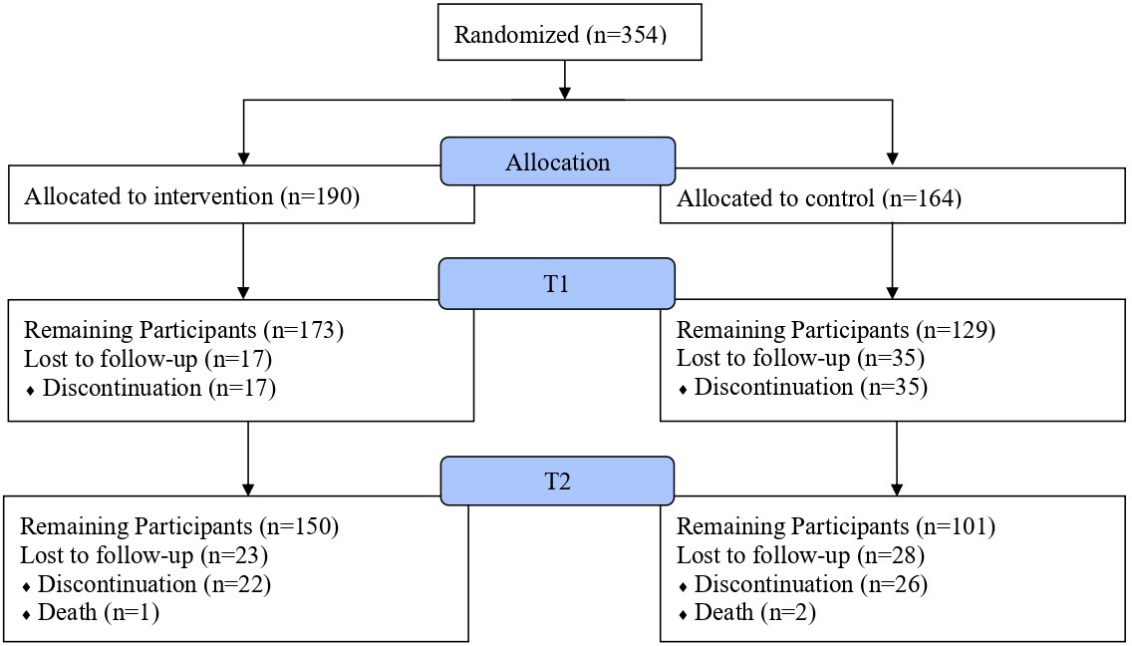
Participant flowchart.

The flow of participants is illustrated in Figure 1. A total of 354 participants were enrolled in the study and either randomized to the intervention group (n=190) or the control group (n=164). In the end, 150 (78.9%) of the intervention group patients and 101 (61.6%) of the control group patients completed the study. The participants were 87.0% (308/354) male, and the mean age was 50.66 years (range 31‐60 years). Although 75.8% of the intervention group was male, 100% of the control group was male. The mean age was 50.23 years (range 31-60 years) in the intervention group and 51.16 years (range 38-61 years) in the control group. Detailed patient characteristics by group can be found in Table 2.

**Table 2.** Patient characteristics at baseline.

	Intervention group (n=190), n (%)	Control group (n=164), n (%)
**Family status**		
Single	28 (14.7)	28 (17.1)
Married	116 (61.1)	98 (59.8)
Divorced/separated	43 (22.6)	33 (20.1)
Widowed	3 (1.6)	5 (3)
**Highest education level**		
No school-leaving certificate	7 (3.7)	7 (4.3)
Secondary/elementary school	74 (38.9)	73 (44.5)
Middle maturity	59 (31.1)	38 (23.2)
Polytechnic high school	5 (2.6)	1 (0.6)
Advanced technical certificate	22 (11.6)	23 (14)
University entrance qualification	23 (12.1)	22 (13.4)
**Highest vocational training**		
None	19 (10)	13 (7.9)
Apprenticeship (vocational training in company)	114 (60)	108 (65.9)
Technical school	35 (18.4)	28 (17.1)
University of applied sciences/school of engineering	6 (3.2)	4 (2.4)
University	9 (4.7)	6 (3.7)
Other	7 (3.7)	5 (3)
**Vocation**		
Workers	76 (40)	58 (35.4)
Employees	99 (52.1)	93 (56.7)
Civil servant	1 (0.5)	0 (0)
Self-employed/freelance	14 (7.4)	13 (7.9)

### Evaluation Outcomes

#### Effects on Quality of Life

The primary outcome of disease-specific quality of life measured by HeartQoL showed a significant average increase after 6 months of 7.35 (95% CI 5.15-9.55) points in the intervention group (*t*_189_=6.60, *P*<.001). The results of the ANOVA are demonstrated in Table 3 and indicate improvements over time that are also higher in the intervention group compared to the control group.

**Table 3.** Results of the HeartQoL, a disease-related quality-of-life questionnaire.

Scale and group		Time point (mean score)			Main effects (ANOVA*, P* value)		
		t0	t1	t2	Time	Group	Time x Group
**Overall**					<.001	<.001	<.001
	Intervention group	22.34	31.98	33.22			
	Control group	20.095	24.18	24.27			
**Physical**					<.001	<.001	.003
	Intervention group	15.77	20.89	22.26			
	Control group	14.02	16.57	16.57			
**Emotional**					<.001	<.001	.67
	Intervention group	6.57	11.09	10.96			
	Control gorup	6.07	7.61	7.80			

#### Effects on Cardiovascular Risk Profile

Table 4 shows the results of the ANOVA for the cardiovascular risk profiles, indicating improvements over time that are higher in the intervention compared to the control group.

**Table 4.** Results from Cardiovascular Risk Management (CARRISMA).

Scale and group		Time point (mean score)			Main effects (ANOVA*, P* value)		
		t0	t1	t2	Time	Group	Time × group
**CV-risk** ^**a**^					<.001	<.001	.002
	Intervention group	2.90	1.30	1.31			
	Control group	2.93	2.11	2.29			
**HA-risk** ^**b**^					<.001	<.001	.03
	Intervention group	7.84	2.81	1.85			
	Control group	8.70	5.76	3.32			

a 10-year risk of cardiovascular death (CV).

b 10-year risk for heart attack (HA).

The cardiovascular risk profile showed a significant improvement over the measured time points in the intervention group, that is, the 10-year risk of cardiovascular death (mean −1.59, 95% CI −2.00 to −1.19; *t*_189_=−8.57; *P*<.001) and the 10-year risk of a heart attack (mean −5.03, 95% CI −6.19 to −3.87; *t*_189_=−7.71; *P*<.05) were both significantly lower at the end of the observation period than at the beginning.

The ANOVA showed significant effects for group for the secondary target parameters of total and low-density lipoprotein (LDL) cholesterol and body weight but not for blood pressure (Table 5). For total and LDL cholesterol, the time as well as the time and group interactions were significant, indicating differences over time by group.

**Table 5.** Results for secondary target parameters.

Scale and group		Time point			Main effects (ANOVA*, P* value)		
		t0	t1	t2	Time	Group	Time × group
**Cholesterol (mg/dL), mean value**					.01	.006	.02
	Intervention group	179.17	167.39	165.10			
	Control group	178.91	177.15	179.01			
**Low-density lipoprotein cholesterol (mg/dL), mean value**					.01	<.001	.02
	Intervention group	111.25	99.47	98.89			
	Control group	111.51	109.79	111.68			
**Mean body weight (kg)**					.22	.008	.049
	Intervention group	92.98	88.57	84.86			
	Control group	93.10	92.59	94.58			
**Mean blood pressure (systolic)**					.23	.31	.91
	Intervention group	131.16	130.22	130.20			
	Control group	128.21	129.41	129.39			
**Number of smokers**					.001	.40	.31
	Intervention group	140	26	5			
	Control group	111	19	3			

In both groups, the blood pressure values were already nonpathological (<140 mm Hg) at the beginning of the intervention (t0). On average, they were 131 mm Hg in the intervention group and 128 mm Hg in the control group. These values also did not change significantly or remain constant over time (Table 5).

At the end of rehabilitation, the values for LDL cholesterol were approximately 111 mg/dL in both groups and thus above both the previous (below 100 mg/dL) and current (below 55‐70 mg/dL) recommended range. In the intervention group, the mean value of total cholesterol significantly decreased from t0 to t1 (*t*_189_=−3.95, *P*<.001; Table 5).

The intervention group patients also significantly reduced their body weight by an average of 4.41 kilograms 6 months after the end of rehabilitation (*t*_189_=−2.97, *P*<.001; Table 5).

At the end of rehabilitation, 140 patients in the intervention group and 111 in the control group were smokers. In both groups, the number of smokers significantly decreased over time but this was not more pronounced in one group versus the other (Table 5).

#### Effects on Sociomedical Acquisition Prognosis

The SIBAR was used to record the participants’ current employment status, degree of reduced earning capacity, and pension entitlement.

In the “sociomedical risk of early retirement” scale, no patient achieved a score above 8, meaning that there was no increased risk of early retirement and no need for occupational treatment for any patient at any measurement time. The “sociomedical risk of early retirement” decreased compared to the initial value at the end of rehabilitation (Table 6).

**Table 6.** Results of the SIBAR.^a^

Scale and group		Time point (mean score)			Main effects (ANOVA*, P* value)		
		t0	t1	t2	Time	Group	Time × group
**Sociomedical/risk of early retirement**					<.001	.39	.89
	Intervention group	5.60	4.90	3.01			
	Control group	5.68	5.15	3.37			
**Subjective need for occupation-related treatment**					<.001	.03	.39
	Intervention group	0.41	0.16	0.07			
	Control group	0.45	0.19	0.19			
**Occupational stress**					.04	.67	.95
	Intervention group	0.49	0.48	0.16			
	Control group	0.40	0.48	0.15			
**SIBAR**					<.001	.11	.89
	Intervention group	5.60	4.90	3.01			
	Control group	5.67	5.15	3.37			

a SIBAR: Screening instrument work and occupation.

Over time, in both groups, the “occupational stress” remained almost constantly below the critical value of 1. The “subjective need for occupation-related treatment” decreased in the first 6 months in both groups and in the intervention group even further until t2.

The total SIBAR score decreased significantly from t0 to t1 (*t*_189_=−2.91, *P*<.05) by on average −0.28 (95% CI −0.48 to −0.09) in the intervention group. However, the ANOVA showed equal reductions in the control group; thus, there was no group effect, only a time effect (Table 6).

## Discussion

### Principal Results

#### Overview

This study showed the potential of an app/web-based cardiovascular rehabilitation program. Patients using the program after leaving the rehabilitation clinic showed significant improvements in both primary endpoints (disease-related quality of life and body weight) compared to control care. Further, the intervention group showed significant improvements compared to the control group in the cardiological risk profile and employment progression.

#### Quality of Life

The main target parameter, disease-related quality of life, was used to determine the extent to which everyday activities as well as physical and emotional functioning were influenced by CHD. At the time of measurement t0 (end of rehabilitation), all patients already had relatively high quality-of-life values since the assessment tool refers to the past 4 weeks. At the time of measurement t0, the patients were at the end of their rehabilitation treatment, during which they were released from daily work, family obligations, and everyday activities in order to recover and focus on themselves and their recovery. At follow-up, the improvement in disease-specific quality of life was found to be clinically significant and more pronounced in the intervention group compared to the control group. The results are in line with another telehealth intervention during and after rehabilitation also showing significant improvements in health-related quality of life in the intervention group (also compared to the control group) [20]. In this study, the improved quality of life is likely driven by the improvement in physical quality of life. In fact, previous studies have shown the positive lifestyle impact, for example, on exercise behavior or dietary habits, when patients follow an additional lifestyle maintenance program after leaving the rehabilitation clinic [20-22]. Apart from modules directly addressing nutrition and exercise, mebix is likely to have improved participants’ understanding of the disease through the knowledge imparted in the program. Similar to other studies, it increased the patients’ awareness of risk and preventive factors for CVDs [23]. Overall, these results are in line with current guidelines and suggestions that emphasize the importance of follow-up care after rehabilitation to maintain the positive health and behavioral effects achieved [24,25].

#### Cardiovascular Risk Profile

The second main target parameter, the cardiovascular risk profile (10-year risk of cardiovascular death and 10-year risk of myocardial infarction) of the patients, was calculated with a special software and considered lifestyle factors such as obesity or cigarette use, both having an important additional prognostic significance. People who are not yet in the treatment-required range with the conventional scores have a significantly higher risk of CVD when obesity and heavy cigarette consumption are considered. In the CARRISMA program, this effect was considered. The results of this study showed a significant improvement in the 10-year risk of cardiovascular death and 10-year risk of myocardial infarction over the measurement time points in the intervention group. Moreover, even though the control group also lowered their risk profiles over time, patients in the intervention group on average decreased their risk profiles more.

Factors explaining this result are certainly the smoking cessation carried out or initiated in the clinic (final point method), the significant weight loss, and the stabilization of a good blood pressure in both groups, as well as the significant improvement in blood lipid values, especially in the intervention group. A systematic review on digital health interventions for cardiovascular rehabilitation also showed significant weight loss in most studies, while results for endpoints were more sparsely reported and more heterogeneous [16]. In fact, several studies did not show favorable results regarding blood lipids [20,21]. This might also be explained by the different focus of the follow-up care provided, with mebix emphasizing nutrition as well as exercise, thus fulfilling critical requirements of a multidisciplinary approach needed for cardiac rehabilitation [25].

In this study, the number of smokers decreased over time in both groups. At the end of rehabilitation, 140 patients in the intervention group and 111 in the control group were smokers. Six months after the end of rehabilitation, the number of smokers decreased to 26 and 19 smokers, respectively. Another 6 months later (ie, 12 months after the end of rehabilitation), only 5 intervention group patients and 3 control group patients still smoked. When assessing the drastic reduction in the number of smokers among the patients, it must be considered that the figures are based on patient data; response behavior under conditions of social desirability must also be considered. However, the results are in line with a study showing that most patients lack knowledge about risk factors such as smoking, stressing the importance of increasing awareness about primary and secondary prevention [23]. The absence of group differences when it comes to smoking and the presence of group differences when it comes to other endpoints influenced by lifestyle choices like exercise and nutrition might be explained by the fact that the latter two are easier to improve and adhere to with additional support after rehabilitation. As such, programs that help patients immediately after leaving rehabilitation can help maintain and further improve the effects of rehabilitation, even in the long term. As a result, digital tools like mebix hold great potential to significantly improve the lifestyle-based recovery dynamics following rehabilitation that are observed in the control group.

#### Need for Occupational Treatment Services

With the help of the SIBAR, it can be shown whether there is an increased sociomedical risk of early retirement and how stressful the patient perceives his or her occupational situation. In all 3 subscales, as well as in the overall assessment, a significant reduction in both groups was present and no group differences could be assumed. In addition, a need for occupation-related treatment was not present for any patients at any time of the observation.

### Strengths and Limitations

This study represents an investigation within health care research that considers new technologies and can also provide viable follow-up benefits for the pension system. The study was conducted under largely real-life conditions. Therefore, the study presents results that are achieved with a concept that could be transferred to the German rehabilitation landscape. However, due to several limitations of the studied patient population, the results are not representative to the general population. First, this study has a gender bias, with 87% of participants being male. Although not being representative of the general public, similarly high percentages of males are present in comparable studies [20-22,26] and there is a higher prevalence of coronary diseases in males [2]. Second, the population is generally younger than most cardiovascular surgical patients (in other telemedicine rehabilitation interventions) [2,3,20], also due to the design and inclusion criteria defined in this study. In this respect, the results show good effectiveness in (younger) men. Third, the population has a low socioeconomic status on average, which increases the risk and need for training. However, the prevalence of coronary disease is highest in people with lower socioeconomic status [2]. Future studies with an even distribution of gender and the inclusion of older patients and patients with higher socioeconomic status must show whether the results can be generalized to both genders, all age groups, and different socioeconomic backgrounds.

The lack of blinding is a major limitation of most digital intervention trials [26] and might have further introduced a performance bias. Additionally, the risk of selection bias cannot be excluded, as motivated patients who are interested in using digital programs are more likely to participate in this study. This bias is likely to exist in other evaluation studies of digital interventions as well, making comparisons between study results more reliable.

Another strength of the study is a relatively large sample size compared to other studies [16,26], even though fewer participants than originally planned were recruited. However, the percentage of patients that dropped out of the intervention group was lower than expected (21%), indicating good adherence and acceptance of the intervention. The originally assumed dropout rate of 40% was observed in the control group. These group differences are comparable to other studies, showing greater adherence in the groups receiving digital interventions compared to traditional care [16]. It is likely that the patients using mebix might have been more motivated to report outcomes compared to the control group patients, potentially translating into systematic differences between patients dropping out and those continuing with the study. In the intervention group, it cannot be excluded that dropouts might have also been connected to dissatisfaction or technical problems with the intervention, as reported in other studies [20].

Although the 2 scores used as secondary endpoints in this study (ie, CARRISMA and SIBAR) are assessment instruments in primary care, they focus on secondary prevention, for which no other tool exists.

### Conclusions

The results of the study indicated a positive effect of an app/web-based intervention as follow-up care for patients leaving a cardiovascular rehabilitation clinic. As cost-efficient and time- and location-independent tools, digital interventions have the potential to extend rehabilitation for up to 12 months outside the clinic and further improve quality of life, cardiovascular risk profiles, and employment prognosis.

## Supplementary material

10.2196/57960 Multimedia Appendix 1 Home screen of the app showing the activity, step, and nutrition tracking, as well as disease-related appointments and to do list (left) or the education features (video, information, calendar; right).

10.2196/57960 Checklist 1 CONSORT-EHEALTH checklist (V1.6.1).

## Abbreviations

CARRISMA: Cardiovascular Risk Management
CHD: coronary heart disease
COPD: chronic obstructive pulmonary disease
CVD: cardiovascular disease
DGPR: German Society for Prevention and Rehabilitation of Cardiovascular Diseases
EHIS: European Health Interview Survey
ESC: European Society of Cardiology
GEDA: Gesundheit in Deutschland aktuell
LDL: low-density lipoprotein
PROCAM: Prospective Cardiovascular Münster
SIBAR: Screening instrument work and occupation
