# Correction: Results of a Digital Multimodal Motivational and Educational Program as Follow-Up Care for Former Cardiac Rehabilitation Patients: Randomized Controlled Trial

**DOI:** 10.2196/73890

**Published:** 2025-05-12

**Authors:** Maxi Pia Bretschneider, Wolfgang Mayer-Berger, Jens Weine, Lena Roth, Peter E H Schwarz, Franz Petermann

**Affiliations:** 1 Department for Prevention and Care of Diabetes, Department of Medicine III, Faculty of Medicine Carl Gustav Carus, Technische Universität Dresden, Fetscherstraße 74, Dresden, 01307, Germany, 49 351 458-2715; 2 Klinik Roderbirken der Deutschen Rentenversicherung Rheinland, Leichlingen, Germany; 3 Vision2B GmbH, Erfurt, Germany

In “Results of a Digital Multimodal Motivational and Educational Program as Follow-Up Care for Former Cardiac Rehabilitation Patients: Randomized Controlled Trial” (JMIR Cardio 2024;8:e57960) the authors noted two errors.

In the Results section of the Abstract, the following sentence:

*Positive effects on secondary outcomes like body weight, blood pressure, and number of smokers only showed time effects, indicating no difference between the groups.*

Has been revised to:

*Secondary outcomes like the body weight and cholesterol levels were significantly reduced in the intervention group, also in comparison with the control group.*

In addition, the degree for author Maxi Pia Bretschneider was removed as it was reported erroneously.

The correction will appear in the online version of the paper on the JMIR Publications website, together with the publication of this correction notice. Because this was made after submission to PubMed, PubMed Central, and other full-text repositories, the corrected article has also been resubmitted to those repositories.

