# Supplementary figures and images for: Results of a Digital Multimodal Motivational and Educational Program as Follow-Up Care for Former Cardiac Rehabilitation Patients: Randomized Controlled Trial
Source: JMIR Cardio. 2024 Dec 11;8:e57960. doi: 10.2196/57960 (PMC11653970; doi:10.2196/57960)

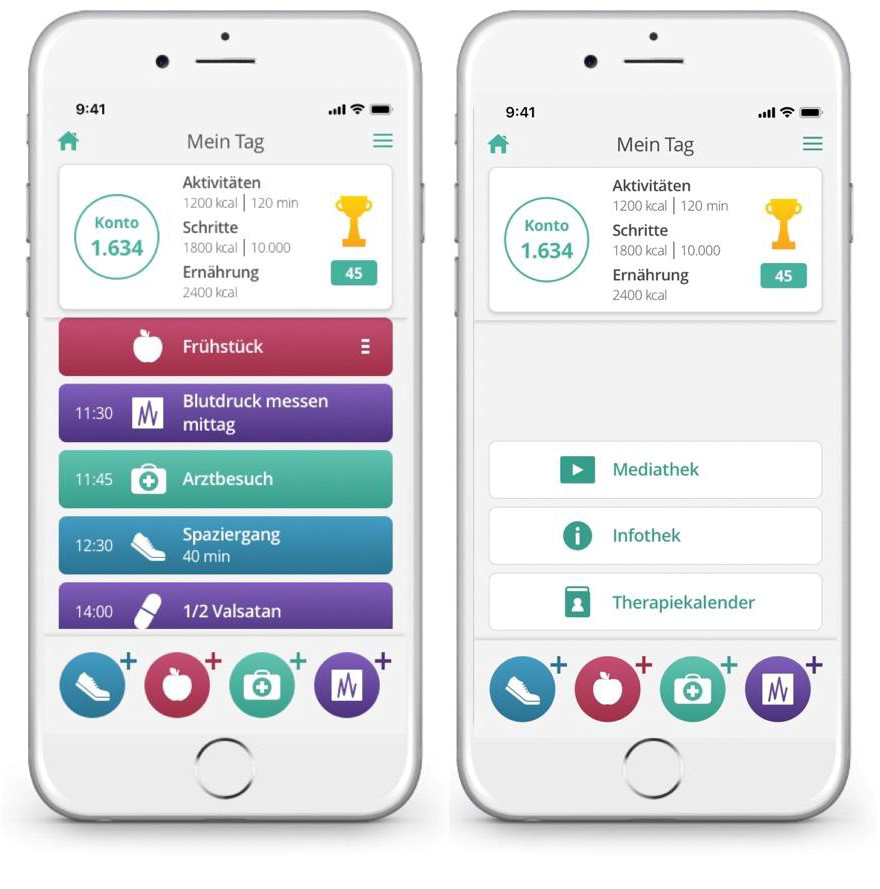

Supplement: Multimedia Appendix 1 [file cardio-v8-e57960-s001.png]
